# The Effect of Arm Dominance on Knee Joint Biomechanics During Basketball Block Shot Single-Leg Landing

**DOI:** 10.2478/hukin-2022-0100

**Published:** 2022-09-08

**Authors:** Parunchaya Jamkrajang, Atipong Mongkolpichayaruk, Weerawat Limroongreungrat, Huw Wiltshire, Gareth Irwin

**Affiliations:** 1 College of Sports Science and Technology, Mahidol University, Salaya Thailand; 2 Cardiff School of Sport and Health Sciences, Cardiff Metropolitan University, Cardiff UK

**Keywords:** anterior cruciate ligament injury, arm movement, basketball, knee joint biomechanics

## Abstract

Single arm blocking is a key component of successful basketball defence. The player uses either their dominant or non-dominant arm to block the ball landing on a common leg. Understanding how the bio-physical loads of the landing leg change as a function of the blocking arm will provide insights into potential injury risk of the lower limb. The aim of this study was to investigate the effects of arm dominance on the biomechanical variables of injury risk of the lower limb, specifically the knee joint during the single-leg landing in female basketball players. Kinematic and kinetic data were collected from fourteen female basketball athletes (20.85 ± 1.35 years, 1.69 ± 0.06 m, 60.37 ± 7.75 kg), each performing three trials of a dominant arm and non-dominant block jump landing on the dominant leg. The results showed significantly higher anterior and medial ground reaction force, knee joint flexion and abduction and lateral knee force during the dominant arm landing (p < 0.05). These findings highlight potential injury risk and the need for the player to be more proficient at dominant arm block-shot landing. The player should aim to develop a larger landscape of technique to meet the demands of the game and facilitate a more effective and safer landing strategy.

## Introduction

Basketball is a globally popular sport with approximately 450 million players participating. Due to the dynamic nature of the game there are high numbers of lower limb injury incidences (Schiltz et al., 2009), particularly in female athletes (Leppänen et al., 2017). The knee is a key site for injury in basketball and there are several factors that contribute to the nature and severity of the injury, i.e. anatomical structure, muscle imbalance and poor landing technique. Landing on one leg is frequently performed in many sports including handball, volleyball and basketball (Ameer et al., 2016; Mall et al., 2014). For basketball, a single-leg landing is a common movement during shooting and blocking. To block a basketball shot, a defensive player often runs or jumps toward an opponent while raising their arm to prevent the shot. If a player jumps toward an opponent, the player needs to control their body in order to avoid a foul. In this situation, athletes may encounter difficulty in maintaining control and stability whilst landing on a single leg (Sugiyama et al., 2014).

Single-leg landings expose the performer to higher bio-physical loads at the knee joint, due to increased vertical ground reaction force and tibial anterior shear force compared with double-leg landings (Wang, 2011; Yeow et al., 2011). Single leg landings have been recognised for their increased injury risk, for example during hopping and landing it was observed that the loading and kinematics of the knee joint were such that the risk of ACL injury was increased, and this was shown to be greater in female athletes (Alentorn-Geli et al., 2009; Sinclair et al., 2019). It is clear from previous research that injury risk of single-leg landings is implicated in the contribution to

the greater risk of non-contact ACL injuries compared to double-leg landings (Boden et al., 2009; Yeow et al., 2011).

The block shot is based on the recognition of this skill being a key factor in the defensive play of basketball (Adkins et al., 2007). The injury rates across the whole sport show females have a higher incidence of lower limb injuries (Cumps et al., 2007; Zelisko et al., 1982) and these are reported to occur from landings such as block shots. More generally, landings are performed frequently in sport activities such as soccer, basketball and volleyball and they have been previously confirmed to be a risk factor for the incidence of non-contact ACL injury (Amraee et al., 2017; Yu et al., 2006).

Basketball block shots are characterised by players disrupting the opponent with their arm and depending on the playing situation will use either the dominant or the non-dominant side (Adkins et al., 2007). There is paucity of research that has examined the influence of the arm selection on the aerial phase and the subsequent landing. Providing knowledge of the landing strategy used and potential injury risk factors would be beneficial to the coaches, clinicians and players. Previous research has highlighted that the motion of arm influences knee joint loading during a single leg landing, for example Masters et al. (2016) investigated positions of the upper limbs during a single leg drop landing. The results demonstrated decreased peak hip flexion and increased ankle dorsiflexion when athletes performed drop landings with arms holding an object on the opposite side with the leg landing (Masters et al., 2016). Furthermore, Chaudhari et al. (2005) found that holding a lacrosse stick in front and holding a ball in the lateral side during side-cutting caused an increased valgus moment during landing. Based on these previous studies, injury risk factors of the knee joint include lesions to the ACL including knee joint motion (flexion, valgus, internal rotation) and knee loading (internal knee forces and moments) during unpredictable landing tasks.

The block jump in basketball provides a unique example of a single leg landing being influenced by arm dominance. Landing tasks are frequently performed in basketball, and there is a wealth of research that has examined the biomechanics of landing across various sports in order to identify injury risks. Examples include gymnastics (Marshall et al., 2007), volleyball (Sinsurin et al., 2017) and stop landing tasks (Yu et al., 2006). Currently, there is a lack of research focused on landing post blocking in basketball and with this being a fundamental part of the game, particularly from a defensive perspective, greater understanding of the potential injury risks in this skill would provide missing information related to the biomechanical demands of this task. Understanding the control of landing based on theories related to biomechanics and movement control is necessary (Goodman et al., 1985; Newell and van Emmerik, 1989; Newell and Vaillancourt, 2001). Whilst the focus of this paper is on the biophysical demands players experience, it is important to consider the concepts of dynamical changes in motor behaviour during these tasks.

Understanding the biomechanical responses of landing will build on previous research which has recognised that the upper limb position affects the lower limb joint biomechanics (Chaudhari et al., 2005; Masters et al., 2016). The current study will provide meaningful information regarding the biomechanics of this skill informing scientists, coaches and clinicians with specific reference to the basketball block. In addition, the paper represents one of the first projects investigating the effect of arms motion in a simulated basketball block.

Therefore, the aim of this study was to examine the effects of dominant and non-dominant arms during a simulated block shot on knee joint biomechanics during a single-leg landing by female basketball players. Our hypothesis was that there would be differences between dominant and non-dominant arm movements in the knee joint biomechanics during a single-leg landing in basketball players and this would have implications in terms of injury risk at the knee joint.

## Methods

### Participants

Fourteen female basketball athletes purposefully sampled, age 20.85 ± 1.35 years, body height 1.69 ± 0.06 m, body mass 60.37 ± 7.75 kg, participated in this study. In our study, leg dominance was evaluated by the single leg hop (furthest distance indicating the dominant leg) and via a questionnaire (asking which leg participants use to kick a ball). Arm dominance was determined via handgrip strength using a handheld dynamometer. The majority of participants were right arm and right leg dominant possessing the same arm and leg dominance (only two players were left arm and left leg dominant). Participants were only included in this study if they were members of the University basketball team, training regularly at least 3 times per week and had no injury history in lower extremity within 3 months. All participants signed a consent form of Siriraj Hospital Human Research Protection before taking part in the study (COA No. Si 435/2018).

### Data Collection

**Figure 1:**
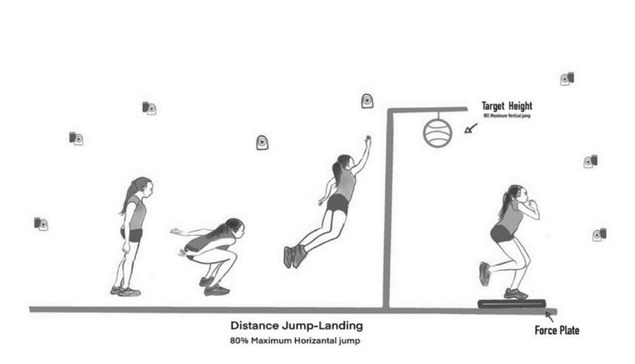
Illustration of the data collection, experimental set up.

Sixty-one reflective markers were placed on the upper body of participants based on the plug-in gait model (Van Rossom et al., 2017) and the lower body using the Liverpool John Moores University marker model (Vanrenterghem et al., 2010). Kinematic (8 motion analysis cameras, RAPTOR-E, 200 Hz) and kinetic (BP400600 AMTI, 2000 Hz) data were collected. Participants performed a self-selected warm-up for 10 minutes before being asked to perform a maximal forward jump with both legs to a target while raising the arm as high as possible, similar to blocking motion in a basketball game. All participants landed onto a force plate (Kistler Instrument, AG, Winterthur, Switzerland) with their dominant leg whilst performing a block with their dominant arm (DA) or the non-dominate arm (NDA). In order to determine the height of the suspended ball and the distance from the take-off point which was used to simulate the block shot, each participant performed a maximum vertical counter movement jump and a maximum horizontal standing broad jump on three separate occasions. 80% of the average of the three maximum vertical and horizontal jump distances were used to define the ball height and horizontal distance from the take-off mark. The data collection set up is illustrated in Figure 1.

### Data Analysis

Cortex Motion Analysis software was used for handling all phases of motion. Furthermore, Cortex software was used to track markers and interpolate missing data. Visual 3D software (C-motion, version 6) was used to analyse kinematic and kinetic data consisting of the three dimensions: GRFs, the knee joint angles, net moments, and internal forces. The initial contact (IC) was defined by the force plate data as higher than 20 N (Lee et al., 2018) and time to the peak knee flexion (PKF) was normalized to 100% of the landing phases. Kinematic and kinetic data were analysed on events following initial contact, the peak vertical ground reaction force (PvGRF) and the PKF. All marker trajectories were filtered at 9 Hz by a Butterworth low pass filter from the location of the three-dimensional coordinates of the markers placed on the body (Kristianslund et al., 2012). GRFs and knee joint forces were normalized to body weight. The knee joint moment was calculated via inverse dynamics in Visual 3D. Variables associated with injuries were selected based on previous research (e.g., Yu et al., 2006) and included: peak GRF (vertical, anterior posterior, and medial lateral), anterior posterior and medial lateral force at peak vertical GRF, GRF at peak knee flexion (vertical, anterior posterior, and medial lateral), the maximum knee angle during landing (flexion/extension, valgus/varus, internal/external rotation), the maximum knee angle during landing at peak vertical GRF, peak knee force, knee force at peak GRF and knee force at peak knee flexion (anterior posterior, medial lateral and compressive), peak knee moment, knee moment at peak GRF and knee moment at peak knee flexion (flexion/extension, valgus/varus).

### Statistical Analyses

All statistics captured were analysed by IBM SPSS Statistics 23.0 software. The data were checked for normality assumptions using the Shapiro-Wilk test. Paired sample *t*-tests were used to test differences of all the data between two landing conditions (NDA and DA). Cohen’s *d* effect size of 0.2 was considered as a ʹsmallʹ effect size, 0.5 represented a ʹmediumʹ effect size and 0.8 represented a ʹlargeʹ effect size (Cohen, 1992). The level of significance was set at *p* < 0.05.

## Results

**Figure 2:**
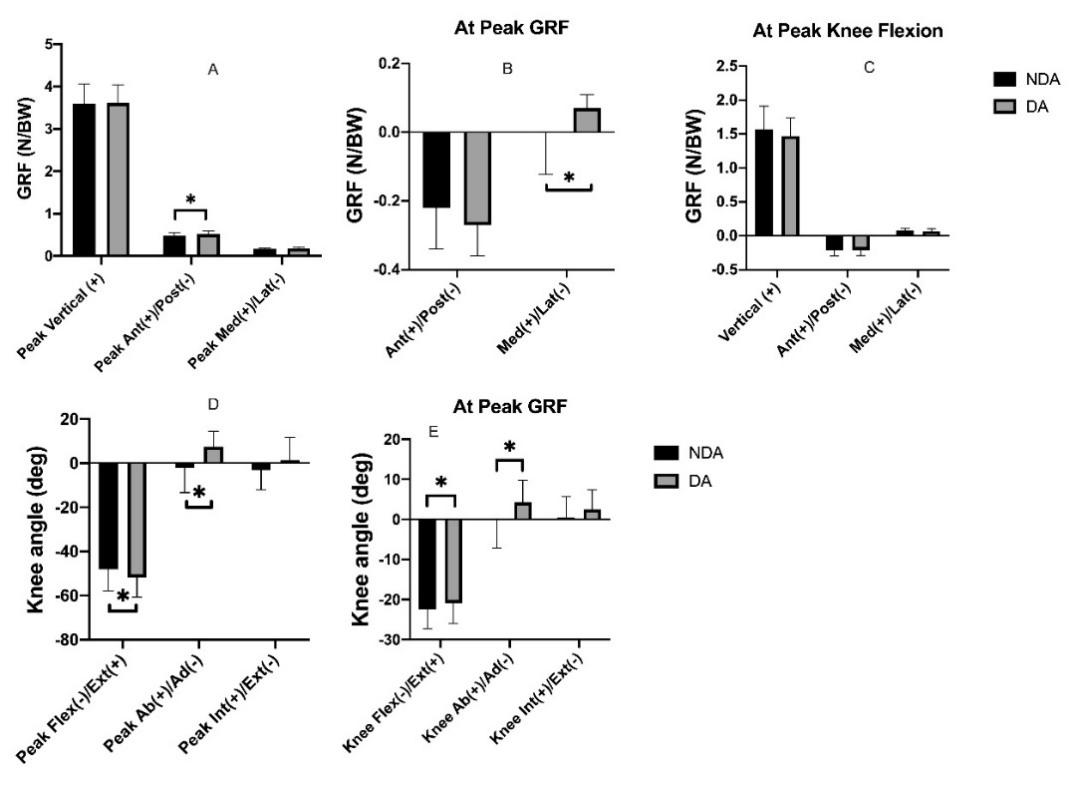
Mean and standard deviation GRF in three axes between NDA and DA landing conditions (A = peak forces, B = anterior-posterior and medial-lateral force at peak vertical force, C = vertical, medial-lateral and anterior-posterior force at maximum knee flexion, D = knee joint angle at peak GRFs in three axes, E = knee joint angle at peak GRFs in three axes).

Significant differences between NDA and DA conditions were observed for anterior GRF (*p* = 0.03, Cohen’s *d* = 0.48). In addition, significant differences between NDA and DA conditions at peak GRF were found in the M/L direction (*p* = 0.04, Cohen’s *d* = 0.35), interestingly, the result showed that in the M/L direction players using the DA strategy were more medial compared to players using the NDA blocking method (Figure 2).

During landing the results revealed significant differences in peak knee flexion between NDA and DA conditions (*p* = 0.04, Cohen’s *d* = 0.62), with the NDA condition showing the smallest knee flexion. Knee abduction (valgus) also showed a significant difference between NDA and DA conditions (*p* = 0.02, Cohen’s *d* = 0.74), with DA players showing abduction compared to the adduction of NDA players (Figure 2). At peak GRF, there were significant differences between NDA and DA conditions for knee flexion (*p* = 0.03, Cohen’s *d* = 0.65) and abduction/adduction (*p* = 0.04, Cohen’s *d* = 0.62), where NDA players demonstrated greater flexion and adduction compared to DA players (Figure 2).

**Figure 3:**
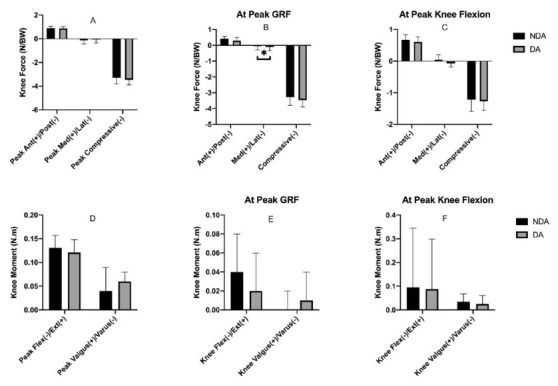
Mean and standard deviation of the knee joint kinetics between NDA and DA landing conditions (A = peak knee forces, B = peak knee forces at peak vertical force, C = peak knee forces at maximum knee flexion, D = peak knee moments, E = peak knee moments at peak vertical force, F = peak knee moments at maximum knee flexion).

The results showed only knee force at peak GRFs in lateral and medial direction (Figure 3) were significantly different between the two conditions (*p* = 0.03, Cohen’s *d* = 0.73), with DA condition showing more lateral force. Figure 3 illustrates the knee joint moments between NDA and DA groups. The results showed that there were no significant differences between these blocking strategies across all joint kinetics. However, there were large levels of participants’ variability shown particularly in the knee flexion/extension moment at peak knee flexion.

## Discussion

Landing on the same leg regardless of the arm used to block occurs in the competitive game of basketball. Understanding the influence of upper limb motion on landing strategy provides meaningful information about the potential injury risk to the lower limb particularly when the landing leg remains the same. The purpose of this study was to examine the effects of dominant (DA) and non-dominant arm (NDA) motions on the biomechanics of the knee joint during the single-leg landing (on the dominant leg) in a simulated basketball block shot. This study revealed significant differences between the motion of the NDA and the DA on landing strategies specifically in terms of GRFs, knee joint angles, and internal knee forces.

Landing from a DA block showed significantly increased peak anterior GRFs, greater medial GRFs at the instant of peak vertical GRFs, greater peak knee angles (flexion and valgus), increased knee valgus at the instant of peak vertical GRFs and peak lateral internal knee joint force at the instant of peak vertical GRFs. The anterior GRF has been associated with injury previously for a landing task (e.g., Yu et al., 2006). In contrast the NDA landing showed greater knee flexion at the instant of peak vertical GRFs, however, a smaller peak knee flexion angle compared to the DA. These findings highlight the mechanical differences between NDA and DA blocking and landing on the same landing leg.

The hypothesis of this study stated that we expected to see differences in the landing strategies, and that there would be implications to injury of the lower limb, specifically the knee. The results are in agreement with our hypothesis in terms of differences between the landing strategies, however, interestingly, landing using the DA technique was associated with more injury risk factors in terms of joint kinetics and external forces, although a less stiff landing also occurred with the NDA (Figure 2). The findings showed no significant difference in peak vertical GRFs between DA and NDA conditions, however, the range of motion at the knee joint suggested a stiffer landing when the block was performed with the NDA due to the reduced knee flexion range of motion (Figure 2). Basketball coaches (e.g., Adkins et al., 2007) advise landing on both legs to prevent asymmetries which may make a difference when blocking as the NDA and the DA create different landing techniques due to blocking with the NDA not being well practiced.

Furthermore, DA landing showed increased anterior GRFs which may be due to the participants being familiar with blocking a shot with their dominant arm. During the DA landing knee flexion increased to absorb impact forces and reduce injury risk. Moreover, when the arm on the contralateral side was used to perform the blocking-shot, decreased GRFs in the A/P direction were observed (Figure 2). This discrepancy may be due to the participantʹs aptitude. Nevertheless, it seems that most athletes are familiar with using a dominant arm to block a shot rather than a non-dominant arm. The effect of the generated GRFs during a jump-landing showed that the lower body worked as a kinematic chain for dissipating an increasing force. Forces are influenced by the difference of movement and knee motion within this chain. For example, a different jump-landing technique can increase GRFs and valgus strain on the knee joint during landing and will decrease the absorption of the forces generated upon landing (Ford et al., 2003; Herrington et al., 2011; McCarthy et al., 2013).

Changes in knee kinematics and kinetics are important variables for landing in terms of injury and performance. Our results showed that the DA condition produced significantly more knee flexion and abduction (knee valgus) than the NDA condition. This may be due to the center of mass (CoM) location shifting to the dominant side and the body compensating in an attempt to maintain stability. The knee angle resulting from knee abduction facilitates the absorption of the load on impact. On the other hand, the NDA condition showed knee adduction (knee varus) which is a consequence of CoM location shifts. At peak GRF, knee flexion is greater in the NDA than in the DA because the CoM location may be on the medial side of the body and also more knee adduction occurs which may cause an injury. Therefore, the body position is less able to attenuate the impact of landing through working muscles crossing the knee joint which hinders the ability to maintain balance. During the peak GRFs, the DA condition showed less knee flexion and more abduction than the NDA condition, thus it may be related to common injuries such as knee injury and ankle sprain (Hewett et al., 2005; Leppänen et al., 2017; Sinsurin et al., 2017). The reduced knee flexion and accompanying increased knee abduction have been previously highlighted as injury risk factors due to the increased strain on the ACL (Hewett et al., 2005). Previous research has suggested that athletes should perform a block shot by increased knee flexion, increased hip flexion and increased ankle dorsiflexion to reduce the magnitude of GRFs and decrease stress at the knee joint (Dai et al., 2015). The current study’s findings are in contrast to previous research that did not find significant differences between knee flexion and valgus angles (Masters et al., 2016).

Increased valgus movement and knee extension are believed to contribute to increased stress on the ACL and predispose an individual to sustaining an ACL injury (Herrington et al., 2011; Mall et al., 2014; Sugiyama et al., 2014; Zahradnik et al., 2014). Research has suggested that increased knee flexion may allow for a decreased knee injury risk as it may put the hamstrings in a more advantageous position to reduce ACL strain (Walsh et al., 2012).

The data supports the notion that knee loading may be influenced by the arm motion and changes in the landing strategy may be induced by positioning of the upper extremity. These observations are supported by previous research that has examined lower limb mechanics and reported differences when landing with arms away from the landing-side limb (Masters et al., 2016). In addition, Chaudhari et al. (2005) highlighted that upper motion can have an influence on the organization of upper limb motion on the knee loading during landing, during a cutting activity. They found a significant decrease in maximum hip flexion and a significant increase in peak dorsiflexion when landing with arms away from the limb-side landing which increased the possibility of ACL injury (Chaudhari et al., 2005). However, Chaudhari et al. (2005) examined a different task focusing on the general idea of the relationship between the upper body motions influencing the landing strategy. As such in the current study athletes used the DA to block the ball even though the NDA was closer to the ball and hence increased the risk of an ACL injury.

The effects of the blocking-shot movement on the knee kinetics may have an influence on ACL injuries. Knee valgus forces were observed to be significantly greater during the DA landing, suggesting a potential injury risk in combination with the increased anterior GRFs, reduced knee flexion and increased knee abduction, placing stress on the ACL. As stated by Smith et al. (2012) the combination of risk factors and their interaction is a factor that may increase the risk of injury. Our hypothesis predicted that there would be differences in knee joint kinetics during single leg landing conditions. The results led us to accept our hypothesis and demonstrated significantly greater peak lateral shear forces in the DA. Previous studies have demonstrated that the anterior shear force on the proximal tibia is an important factor during single-leg landings (Wang, 2011). This may indicate that the direction of knee force tends to follow the direction of arm movement and specifically the DA methods of blocking may expose the performer to injury risk specifically at the knee. The current study focused on landing on the same leg with differing upper limb motions. In terms of future research and limitations of this study, the following are necessary to state. Firstly, the jumping range and velocity were not measured and these variables may be a potential mechanism for injury risk during a single leg landing. In addition, this may have implications in terms of movement control and would make an interesting future study. Secondly, future research could explore the other landing leg and/or various landing strategies. Thirdly, the jump landing task was performed in horizontal and vertical directions, thus, future research could examine side-jump landing and changes in jumping directions. Finally, in order to increase ecological validity future research could include more game specific environments.

## Conclusions

Our findings of greater peak lateral internal knee joint force and knee valgus at peak vertical GRFs with the DA suggest mechanical differences in landing strategy when different arms are used for the block-shot. Thus, there may be potential implications to injury when landing on the dominant leg after jumping with the dominant arm. Therefore, athletes should practice the blocking maneuver with both the DA and NDA to become equally proficient at landing on their dominant leg. These recommendations aim to provide basketball players with a more effective (and safer) landing strategy.
